# A Case of Multicystic Biliary Hamartoma with a Marked Peribiliary Gland Component Successfully Treated by Purely Laparoscopic Anatomical Liver Resection

**DOI:** 10.1007/s12029-022-00893-1

**Published:** 2022-11-30

**Authors:** Keita Kai, Takao Ide, Tomokazu Tanaka, Kumpei Yukimoto, Hiroyuki Irie, Hirokazu Noshiro, Shinichi Aishima

**Affiliations:** 1 Department of Pathology, Saga University Hospital, Nabeshima 5-1-1, Saga, 849-8501 Japan; 2 Department of Surgery, Faculty of Medicine, Saga University, Saga, Japan; 3 Departments of Pathology & Microbiology, Faculty of Medicine, Saga University, Saga, Japan; 4 Department of Radiology, Faculty of Medicine, Saga University, Saga, Japan

**Keywords:** Multicystic biliary hamartoma, Peribiliary gland, Mucinous cystic neoplasm, Biliary adenofibroma, Laparoscopic liver resection, Case report

## Abstract

**Background:**

Multicystic biliary hamartoma (MCBH) is an extremely rare benign liver lesion characterized by a gross well-circumscribed multicystic honeycomb appearance. This report presents a MCBH case with a marked peribiliary gland component which showed unusual histology.

**Case Presentation:**

A 63-year-old Japanese male was admitted to our hospital for a detailed examination of a hepatic cystic lesion, which was originally detected 14 years ago and had slowly enlarged. A preoperative imaging study revealed a well-demarcated multicystic lesion without communication to the biliary tracts. The possible clinical diagnoses were mucinous cystic neoplasm (MCN) or MCBH. The lesion was successfully resected by purely laparoscopic right anterior sectionectomy. The cut surfaces of resected specimens grossly exhibited a well-circumscribed multicystic lesion with a thick septum. Histologically, the cyst wall was covered by cuboidal epithelial cells resembling epithelium of the bile duct while abundant small ducts, which morphologically resembled peribiliary glands, were observed among the fibrous stroma of the thick septum. Although possible pathological diagnosis varied, including intrahepatic cholangiocarcinoma, intraductal papillary neoplasm of the bile duct, biliary adenofibroma, MCN and MCBH, the lesion was finally diagnosed as MCBH with a marked peribiliary gland component.

**Conclusions:**

MCBH can contain abundant peribiliary glands in the fibrous stroma. A pathologist should be careful not to diagnose such peribiliary glands in MCBH as neoplastic glands.

## Background

Multicystic biliary hamartoma (MCBH) is an extremely rare benign liver lesion which was initially defined by Zen et al. in 2006 [1]. The typical characteristics of MCBH are a gross, well-circumscribed, multicystic honeycomb appearance and histological findings of cystic or ductal structures composed of ductal epithelium, with various amounts of peribiliary (periductal) glands and fibrous connective tissue within the lesion [1–6].

We have recently experienced a MCBH case with a marked peribiliary gland component which showed unusual histology as MCBH and needed particular attention for discrimination from mucinous cystic neoplasm (MCN), biliary adenofibroma (BAF), or intrahepatic cholangiocarcinoma. This case was successfully treated by purely laparoscopic anatomical liver resection that involved complex laparoscopic surgical procedures that could only be performed in very few institutions [7].

## Case Presentation

**Fig. 1:**
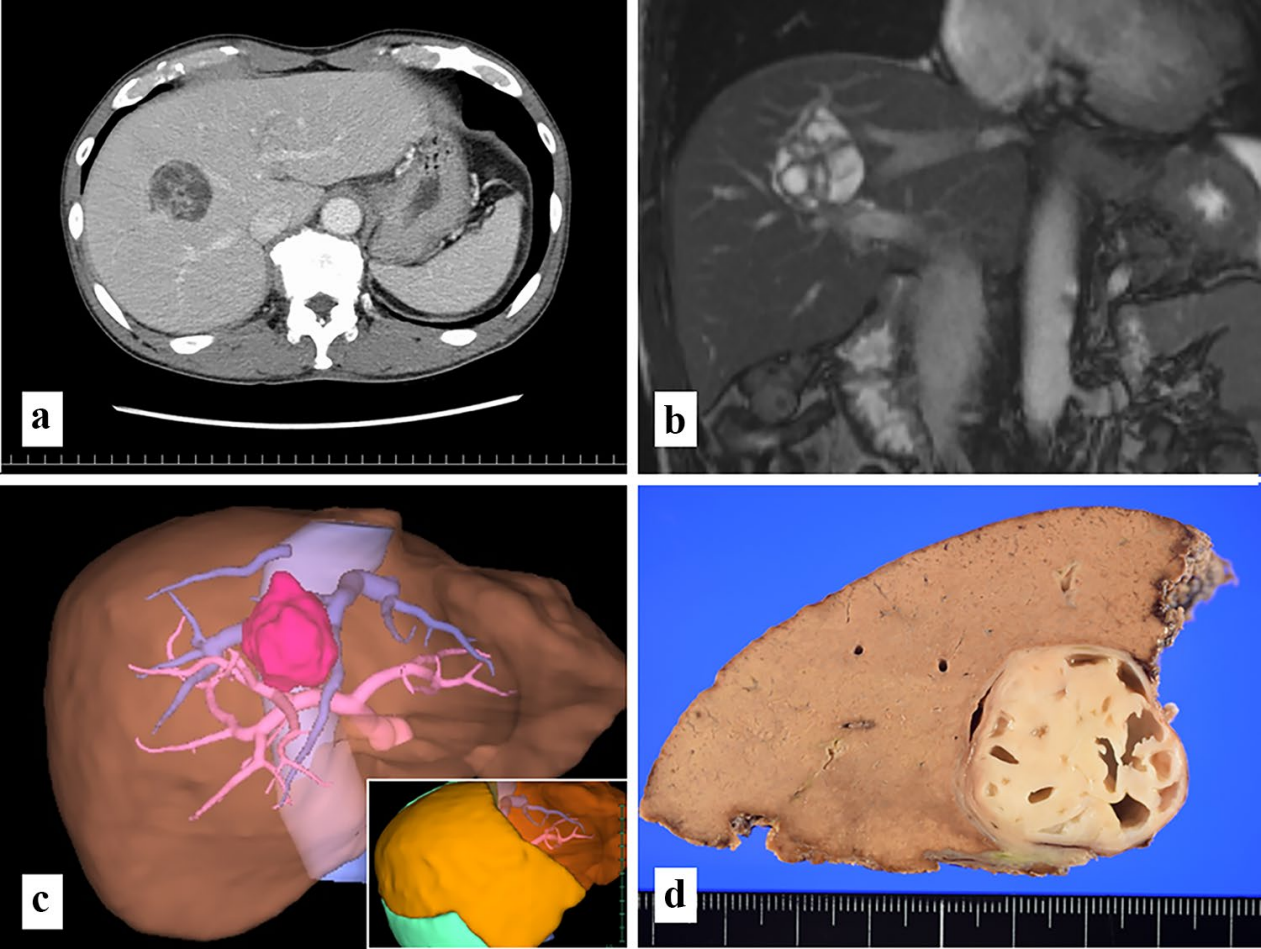
**a** Delayed arterial phase of dynamic CT. A well-demarcated multicystic lesion with a thick septum is shown across segment 8 of the liver. The lesion is slightly enhanced by contrast medium. **b** T2-weighted magnetic resonance image. The lesion was revealed as a multiple cystic lesion of high signal intensity. **c** Three-dimensional reconstruction of abdominal CT scans. The multicystic lesion is represented as a deep pink lesion. Inset is a simulation image of right anterior sectionectomy (total liver volume 1513 mL; resection volume including tumor volume 462 mL; resection rate, 30.5%).** d** Gross appearance of a resected specimen. The lesion was a well-circumscribed multicystic lesion with a thick fibrotic septum

A 63-year-old Japanese male was admitted to our hospital for the detailed examination of a hepatic cystic lesion, which was originally detected 14 years ago and had slowly enlarged (initial detected size was 16 mm in diameter). Laboratory tests including tumor markers showed no remarkable abnormality. Abdominal computed tomography (CT) revealed a well-demarcated multicystic lesion with a thick septum that was 4.2 × 4.1 cm in diameter across segment 8 of the liver (Fig. 1a). T2-weighted magnetic resonance imaging clearly revealed a multicystic lesion with fluid content (Fig. 1b). Endoscopic retrograde cholangiography (ERC) showed that there was no detectable communication between the multicystic lesion to the biliary tracts, and this finding was also confirmed (no remnant contrast medium in multicystic lesion) by abdominal CT scans after ERC.

From these radiological findings, MCN or MCBH were considered as potential clinical diagnoses. Considering the malignant potential, its isolation from the biliary system, anatomical location. and the hepatic functional reserve of the patient, an optimal procedure for surgical resection was discussed. We concluded that right anterior sectionectomy was optimal after simulation using three-dimensional reconstruction of abdominal CT scans (total liver volume, 1513 mL; resection volume including tumor volume, 462 mL; resection rate, 30.5%) (Fig. 1c). Furthermore, we selected pure laparoscopic surgery which is less invasive than conventional open liver resection. The patient successfully underwent purely laparoscopic right anterior sectionectomy (operation time, 435 min; blood loss, 212 g). Intraoperative frozen pathological diagnosis was not performed. The lesion was completely resected, and no recurrence was observed at the time of writing this case report (8 months after surgery).

## Pathological Findings

**Fig. 2:**
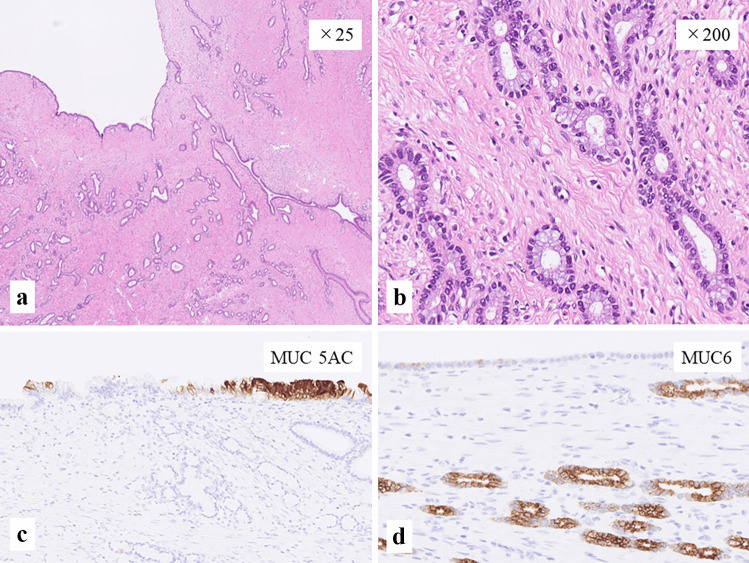
**a** Low magnification (× 25) of the HE-stained specimen. Many small ducts among the fibrous stroma could mimic the invasive growth of adenocarcinoma. The cyst wall was mainly lined by cuboidal epithelium resembling epithelium of the bile duct. Ovarian-like stroma were not observed. **b** At high magnification (× 200), many small ducts in the fibrous stroma morphologically resembled peribiliary glands, lacked the nuclear atypia, and the mitotic figure could not be determined. **c** Immunohistochemistry of MUC 5AC (× 200). The cyst-lined epithelium showed focal positive staining for MUC5AC, while the peribiliary gland-like epithelium showed no positivity for MUC 5AC. **d** Immunohistochemistry of MUC 6 (× 200). The peribiliary gland–like epithelium showed diffuse positivity for MUC 6, while cyst-lined epithelium showed no positive staining for MUC6

The cut surfaces grossly showed a well-circumscribed multicystic lesion with a thick septum (Fig. 1d**)**. Histologically, the cyst wall was covered by cuboidal epithelial cells resembling epithelium of the bile duct and many small ducts resembling peribiliary glands were observed in the fibrous stroma (Fig. 2a). The small ducts lacked nuclear atypia and the mitotic figure could not be determined by observation at high-magnification (Fig. 2b). Thus, the possibility of invasive adenocarcinoma was denied. Possible differential diagnoses for this cystic lesion were intraductal papillary neoplasm of the bile duct (IPNB), MCN, biliary adenofibroma (BAF) or MCBH; however, IPNB was first ruled out because of isolation of the biliary tract.

Ovarian-like stroma, which are necessary for the pathological diagnosis of MCN [8], were not observed even after immunohistochemistry (IHC) to detect estrogen and progesterone receptors and alpha-inhibin. In IHC analyses, both the bile duct–like and peribiliary gland–like epithelium were positive for cytokeratin (CK) 7 and CK 19, but negative for CK 20, MUC 2, and p53. The Ki-67 labeling index was 2% at the hot spot. The bile duct–like epithelia were focally positive for MUC 5AC and MUC 6. Notably, peribiliary gland–like epithelia were diffusely positive for MUC 6 (Fig. 2c, d) and negative for MUC 5AC. This phenotype is consistent with peribiliary glands.

BAF usually presents as a solid and/or sponge-like microcystic gross appearance and glandular structure of biliary-type epithelium [9], and therefore, BAF was not suitable for diagnosis of the present case. From these findings and clinical considerations, we finally diagnosed the case as MBCH with a marked peribiliary gland component.

## Discussion

MBCH was initially reported as a hamartomatous lesion located around the hepatic capsule close to the fissure of the falciform ligament that protrudes from the liver [1]. However, published case reports found that MCBH could also occur deeper within the hepatic parenchyma [2], similar to the present case findings. MBCH is usually composed of ductal structures of biliary-type epithelia and fibrous stroma. Although MCBH usually contains a small amount of peribiliary glands, a marked peribiliary gland component, which was observed in the present case, has not been previously reported. Therefore, we initially hesitated to diagnose the case as MCBH. However, the presence of a marked peribiliary gland component also fits the concept of a hamartoma. In addition, its slow-growing nature over 14 years, isolation from the biliary system, and well-circumscribed gross appearance were all consistent features of a hamartoma.

It is most important that the peribiliary glands in fibrous stroma of MCBH are never diagnosed as an invasive component of MCN or intrahepatic cholangiocarcinoma. Careful observation for cytological atypia, assessment of mitotic activity using Ki-67 IHC, the presence of uniform glands resembling peribiliary glands, and strict differential diagnosis of MCN focusing ovarian-like stroma will prevent misdiagnosis.

In conclusion, we have reported a case of MCBH with an abundant peribiliary gland component that was successfully treated by purely laparoscopic anatomical liver resection. We believe that this case report will be good reference for both pathologists and clinicians alike when they encounter a similar case in the future.

## Data Availability

Data are available on reasonable request from the corresponding author due to privacy or other restrictions.

## Abbreviations

MCBH: multicystic biliary hamartoma
MCN: mucinous cystic neoplasm
BAF: biliary adenofibroma
CT: computed tomography
ERC: endoscopic retrograde cholangiography
IPNB: intraductal papillary neoplasm of the bile duct
BAF: biliary adenofibroma
IHC: immunohistochemistry
CK: cytokeratin
